# Tyrosine 146 of the Human Na^+^/Taurocholate Cotransporting Polypeptide (NTCP) Is Essential for Its Hepatitis B Virus (HBV) Receptor Function and HBV Entry into Hepatocytes

**DOI:** 10.3390/v14061259

**Published:** 2022-06-09

**Authors:** Dariusz Zakrzewicz, Regina Leidolf, Sebastian Kunz, Simon Franz Müller, Anita Neubauer, Silke Leiting, Nora Goldmann, Felix Lehmann, Dieter Glebe, Joachim Geyer

**Affiliations:** 1 Institute of Pharmacology and Toxicology, Faculty of Veterinary Medicine, Justus Liebig University Giessen, 35392 Giessen, Germany; regina.leidolf@vetmed.uni-giessen.de (R.L.); sebastian.kunz@vetmed.uni-giessen.de (S.K.); simonfranzmueller@gmail.com (S.F.M.); anita.m.neubauer@vetmed.uni-giessen.de (A.N.); silke.leiting@vetmed.uni-giessen.de (S.L.); joachim.m.geyer@vetmed.uni-giessen.de (J.G.); 2 Institute of Medical Virology, National Reference Centre for Hepatitis B Viruses and Hepatitis D Viruses, German Center for Infection Research (DZIF, Partner Site Giessen-Marburg-Langen), Justus Liebig University Giessen, 35392 Giessen, Germany; nora.goldmann@viro.med.uni-giessen.de (N.G.); felix.lehmann@viro.med.uni-giessen.de (F.L.); dieter.glebe@viro.med.uni-giessen.de (D.G.)

**Keywords:** bile acid, HBV, infection, NTCP, preS1, receptor, transporter, mutation

## Abstract

Na^+^/taurocholate cotransporting polypeptide (NTCP, gene symbol SLC10A1) is a hepatic bile acid uptake carrier participating in the enterohepatic circulation of bile acids. Apart from its transporter function, NTCP acts as the high-affinity liver-specific receptor for the hepatitis B virus (HBV), which attaches via its preS1-peptide domain of the large surface protein to NTCP, subsequently leading to endocytosis of the virus/NTCP-receptor complex. Although the process of NTCP-dependent HBV infection of hepatocytes has received much attention over the last decade, the precise molecular sites of the virus/NTCP interaction have not been fully identified. Inspection of the primary protein sequence of human NTCP revealed 139YIYSRGIY146 as a highly conserved tyrosine-rich motif. To study the role of Y139, Y141 and Y146 amino acids in NTCP biology, the aforementioned residues were substituted with alanine, phenylalanine or glutamate (mimicking phosphorylation) using site-directed mutagenesis. Similar to wt NTCP, the Y139A, Y141A, Y146A, Y141F, Y146F, and Y146E mutants were expressed at the plasma membrane of HEK293 cells and exhibited intact bile acid transport function. Y146A, Y146E, and Y146F demonstrated transport kinetics comparable to wild-type NTCP with K_m_ values of 57.3–112.4 µM and V_max_ values of 6683–7579 pmol/mg protein/min. Only Y141E was transport deficient, most likely due to an intracellular accumulation of the mutant protein. Most importantly, Y146A and Y146E mutation completely abrogated binding of the viral preS1-peptide to NTCP, while the Y146F mutant of NTCP showed some residual binding competence for preS1. Consequently, the NTCP mutants Y146A and Y146E, when expressed in HepG2 hepatoma cells, showed complete loss of susceptibility for in vitro HBV infection. In conclusion, tyrosine 146, and to some extent tyrosine 141, both belonging to the tyrosine-rich motif 139YIYSRGIY146 of human NTCP, are newly identified amino acid residues that play an essential role in the interaction of HBV with its receptor NTCP and, thus, in the process of virus entry into hepatocytes.

## 1. Introduction

The Na^+^/taurocholate co-transporting polypeptide (NTCP, gene symbol SLC10A1) is the first identified member of the solute carrier family SLC10 [1]. NTCP functions as a bile acid uptake carrier in the sinusoidal membrane of hepatocytes and, together with the apical sodium bile acid transporter (ASBT, gene symbol SLC10A2), which is expressed in the brush border membrane of ileal enterocytes, maintains the enterohepatic circulation of bile acids [2]. Apart from its primary transporter function, NTCP is the high-affinity entry receptor for the hepatitis B virus (HBV) and its satellite virus, the hepatitis D virus (HDV) [3,4]. The virus entry of HBV and HDV involves several steps at the hepatocyte membrane. Among them, the high-affinity virus binding to NTCP, which occurs via the myristoylated preS1-lipopeptide comprising the N-terminal amino acids 2–48 (myr-preS1_2–48_) of the large HBV surface protein, represents the most fundamental step for virus entry [5,6]. Thereby, NTCP significantly accounts for liver tropism and species-specific susceptibility of HBV and HDV infections at the entry level [6]. Since HBV causes 900,000 deaths annually, predominantly due to cirrhosis and hepatocellular carcinoma after chronic hepatitis B [7], novel and more efficient treatment options are desperately needed. Based on this, a deeper understanding of the exact molecular interaction between HBV/HDV and the hepatic receptor NTCP for the development of novel NTCP-directing drugs, that can block virus binding and entry into hepatocytes, is essential [8].

Although NTCP-dependent HBV infection has received much attention over the last decade, the precise molecular sites of the virus/NTCP interaction have not been fully identified [9,10]. Nonetheless, some molecular determinants that are important for virus attachment to NTCP/Ntcps of different species have already been reported [3,4,10,11,12]. For instance, a small motif containing residues 84RLKN87 in human NTCP and 84HLTS87 in mouse Ntcp is essential for HBV infection in humans, but restricts infection in cells expressing the mouse Ntcp. The humanization of this sequence (H84R/T86K/S87N) rescued HBV infection suggesting that R84, K86 and N87, which are localized in the first extracellular loop of human NTCP, are decisive host determinants [4]. Other comprehensive studies proposed the motif 157KGIVISLVL165 as an important binding motif required for the interaction between the viral preS1 domain and NTCP [3,4]. More detailed analyses revealed that the single G158 residue within this fragment was sufficient to discriminate between the HBV/HDV susceptible groups of humans and apes (all bearing G158) and the non-susceptible group of old world monkeys (all bearing R158). Interestingly, G158R mutation of human NTCP completely abolished susceptibility to HBV and HDV infection, while the R158G exchange was sufficient to transmute the old world monkey Ntcp to a functional HBV/HDV receptor [11]. Based on recently published cryo-electron microscopy (EM) structures [13,14,15], NTCP can undergo a conformational transition from an outward- to an inward-facing state, whereby opening of a relatively wide transmembrane pore/tunnel allows substrate translocation from the extracellular to the intracellular compartment. In addition, cryo-EM analysis of preS1-bound NTCP suggested that the myr-preS1 peptide binds preferentially to the open-pore state of the carrier and thereby competes with bile acids for binding to residues lining the same cavity [13,14,15]. This is in agreement with experimental data clearly demonstrating that bile acids, such as taurocholic acid (TC), efficiently block myr-preS1 peptide binding to NTCP [11,16,17]. Furthermore, a single serine residue at the position 267 is involved in the regulation of NTCP activity. The genetic NTCP variant S267F abolished the transporter function of NTCP, at least for bile acids, and made NTCP a less efficient receptor for HBV entry [18]. This finally resulted in decreased susceptibility to HBV and HDV infection in humans expressing this variant with concomitant reduction of advanced stages of HBV-related liver diseases [19,20]. Thus, the S267F “loss-of-function” variant affects the same domain on the NTCP protein that interacts with bile acids and the viral preS1 peptide [13,14,15,17,21].

Apart from these particular NTCP amino acid residues, posttranslational modifications, such as N-glycosylation at N5 and N11 of human NTCP, seem to play a role for the transporter and receptor functions of NTCP. It was demonstrated that N5A/N11A and N5Q/N11Q double mutants lacking both N-glycosylation sites of NTCP were transport negative for bile acids and showed markedly diminished NTCP-driven HBV entry into hepatocytes. As N-glycosylation generally alters the dimerization properties of proteins [22], and NTCP dimerization normally occurs early in the sorting process of the protein towards the plasma membrane [23,24,25], changes in the glycosylation status of NTCP could fundamentally affect its proper sorting to the plasma membrane, where NTCP fulfills its transporter and receptor functions. 

Taken together, based on the current knowledge of relevant NTCP domains that are important for virus binding to NTCP, a very rough map of virus/receptor interaction sites at the surface of NTCP is currently available. Although several independent cryo-EM structures of human NTCP have recently been released that confirmed these previously identified preS1 binding regions at NTCP [13,14,15], the precise molecular interaction sites between NTCP and the myr-preS1 peptide could not be completely determined. Based on this, it is very likely that there are still a number of yet undiscovered surface amino acid residues of NTCP that orchestrate together with the already identified binding regions the HBV/HDV receptor function of NTCP. Hence, in the present study we aimed to find additional NTCP protein regions that are relevant for virus docking to the NTCP receptor based on mutational studies and biochemical analysis. Our research was based on the identification of the highly conserved tyrosine-rich motif 139YIYSRGIY146 of human NTCP that is localized at the junction of transmembrane domain (TMD) 4 and extracellular loop 2.

## 2. Materials and Methods

### 2.1. Chemicals

All the chemicals, unless otherwise stated, were purchased from Sigma-Aldrich (St. Louis, MO, USA). Radio-labelled [^3^H]taurocholic acid ([^3^H]TC, 10 Ci/mmol) was purchased from PerkinElmer Life Sciences (Waltham, MA, USA).

### 2.2. Cell Culture

GripTite HEK293 MSR cells (Thermo Fisher Scientific, Waltham, MA, USA), further referred to as HEK293 cells, were cultured in DMEM (Gibco, Carlsbad, CA, USA) supplemented with 10% fetal calf serum (Pan-Biotech, Aidenbach, Germany), L-glutamine (4 mM, Anprotec, Bruckburg, Germany), penicillin (100 U/mL, Anprotec, Bruckburg, Germany), and streptomycin (100 μg/mL, Anprotec, Bruckburg, Germany) in a 5% CO_2_ atmosphere at 37 °C. T-Rex Flp-In HEK293 cells (Thermo Fisher Scientific, Waltham, MA, USA) were grown in 1:1 DMEM/Ham 12 (Gibco, Carlsbad, CA, USA) supplemented with 10% fetal calf serum (Pan-Biotech, Aidenbach, Germany), L-glutamine (4 mM, Anprotec, Bruckburg, Germany), penicillin (100 U/mL, Anprotec, Bruckburg, Germany), streptomycin (100 μg/mL, Anprotec, Bruckburg, Germany) and 100 µg/mL hygromycin (Carl Roth, Karlsruhe, Germany) in a 5% CO_2_ atmosphere at 37 °C.

### 2.3. Side-Directed Mutagenesis and Cell Transfection

Site-directed mutagenesis was performed on the cloned human NTCP cDNA (according to GeneBank Accession No. NM_003049) in the pcDNA5/FRT/TO vector [26] using oligonucleotide primers synthesized from Metabion International AG (Planegg, Germany). All primers are listed in the Table 1. The generated mutants were sequence-verified by Sanger sequencing (Microsynth AG, Balgach, Switzerland). The constructs were used for transient transfection of HEK293 cells or for the generation of tetracycline inducible T-Rex Flp-In HEK293 NTCP stable cell lines (wild-type NTCP, as well as Y146A, Y146E, and Y146F NTCP mutants). Transfection in all cases was performed using Lipofectamine 2000 according to manufacturer’s instructions (Invitrogen, Carlsbad, CA, USA).

### 2.4. Generation of NTCP-HEK293 Stable Cell Lines

T-Rex Flp-In HEK293T cells (Thermo Fisher Scientific, Waltham, Massachusetts, USA) were used to generate stably transfected tetracycline inducible NTCP-FLAG cell lines as described [16]. Briefly, pcDNA5/FRT/TO wild-type (wt) NTCP, as well as Y146A, Y146E, and Y146F NTCP mutant constructs were co-transfected with the Flp recombinase coding plasmid pOG44 (Invitrogen, Carlsbad, CA, USA). Stable integration of the expression plasmid was achieved through homologous recombination at the Flp recombinase target (FRT) site in the plasmid and cell genome. Stable cell clones were selected for hygromycin (150 µg/mL) resistance for several weeks. The parental cell line and all stable cell clones were maintained at 37 °C, 5% CO_2_, and 95% humidity in DMEM/F-12 medium (PAA, Pasching, Germany) supplemented with 10% fetal calf serum, 4 mM L-glutamine (PAA, Pasching, Germany) and penicillin/streptomycin (PAA, Pasching, Germany). Proper integration of the NTCP mutant constructs into the HEK293 cell genome was verified by Sanger sequencing (Microsynth AG, Balgach, Switzerland).

### 2.5. Western Blotting

HEK293 cells grown in six-well plates were transiently transfected with NTCP-FLAG wt or the respective FLAG-tagged tyrosine mutant constructs. After 48 h, cells were washed with phosphate-buffered saline (PBS; 137 mM NaCl, 2.7 mM KCl, 1.5 mM KH_2_PO_4_, 7.3 mM Na_2_HPO_4_, pH 7.4) prior to being harvested in 500 µL lysis buffer consisting of 20 mM Tris-HCl, 135 mM NaCl, 10% glycerol (all above mentioned reagents from Carl Roth, Karlsruhe, Germany) and 1% Nonidet P40 (Fluka BioChemica, Schwerte, Germany). After centrifugation at 10,000× *g* for 10 min, the supernatants were collected and the amount of protein in each sample was determined using the BCA Protein Assay Kit (Novagen, St. Louis, MO, USA). Hundred µg of proteins were mixed with Laemmli sample buffer containing 2% SDS (Carl Roth, Karlsruhe, Germany), 10% glycerol (Carl Roth, Karlsruhe, Germany), 0.002% bromophenol blue (Merck, Darmstadt, Germany), 62.5 mM Tris-HCl (Carl Roth, Karlsruhe, Germany) and 5% 2-mercaptoethanol (Carl Roth, Karlsruhe, Germany) and heated at 95 °C for 10 min. Then, samples were separated on a 10% SDS polyacrylamide gel under reducing conditions, followed by electro-transfer to a Roti-PVDF membrane (Carl Roth, Karlsruhe, Germany). After blocking in TBS-T buffer (0.05% Tween-20 (Carl Roth, Karlsruhe, Germany) in PBS) containing 5% non-fat milk (Carl Roth, Karlsruhe, Germany), the membranes were probed with anti-FLAG rabbit polyclonal antibody (1:2000, Sigma-Aldrich, Hamburg, Germany, Cat. #F7425) overnight at 4 °C. Staining against beta-actin or GAPDH served as loading controls and were detected using a mouse anti-β-actin (1:1000, Sigma Aldrich, Cat. #A5441) or goat anti-GAPDH antibodies (1:1000, Sigma-Aldrich, Hamburg, Germany, Cat. #SAB2500450). Subsequently, the membranes were incubated with peroxidase-labelled anti-rabbit and anti-mouse goat secondary antibodies (1:3000, all from Thermo Fisher Scientific, Waltham, MA, USA; Cat. #31460, #31430, and #31431, respectively). Membranes were finally incubated with the ECL Plus Kit (Amersham Biosciences, Buckinghamshire, UK) and signal was acquired using the ChemiDoc Image System and Image Lab software (Bio-Rad Laboratories, Hercules, CA, USA).

### 2.6. Bile Acid Transport Assay

Qualitative transport experiments were performed in NTCP-transfected HEK293 cells with the fluorescent bile acid 4-nitrobenzo-2-oxa-1,3-diazole taurocholic acid (NBD-TC) in DMEM [27]. After incubation with taurocholic acid (TC) for 30 min at 37 °C, HEK293 transiently expressing the NTCP-FLAG proteins were washed with PBS. Nuclei were stained with Hoechst33342 (Thermo Fisher Scientific, Cat. #62249) and then TC transport was analyzed. Live-cell images were captured by Leica DMI6000 B inverted fluorescent microscope using a ×20 objective and quantitatively analyzed using the LAS X software (Leica, Wetzlar, Germany). Cell-based fluorescence was determined by defined regions of interest (ROI), and data are presented as the mean background-subtracted fluorescence intensity of empty vector (EV)-transfected HEK293 cells. Quantitative transport measurements were performed with [^3^H]taurocholic acid ([^3^H]TC, 20 Ci/mmoL, 0.09 mCi/mL, Perkin Elmer, Waltham, MA, USA) in HEK293 cells transiently overexpressing NTCP proteins as previously reported [11]. Briefly, cells were seeded into polylysine-coated 24-well plates and transfected with NTCP wt or tyrosine mutant constructs. After 48 h, growth medium was aspirated and each well was rinsed three times with 0.5 mL of incubation buffer (Hanks’ balanced salt solution [HBSS] buffer supplemented with 20 mM HEPES, pH 7.4) and incubated for at least 20 min at 37 °C. The incubation buffer (further referred to as transport buffer) was removed and 200 µL of incubation buffer containing radiolabeled and non-radiolabeled substances was added to each well and incubated at 37 °C for 3 min. After incubation, the uptake was terminated by aspirating the reaction mixture and washing the cells three times with 0.4 mL of ice-cold PBS. Cells were solubilized with 0.6 mL of 1 N NaOH overnight. Cell-associated radioactivity was measured after the addition of 2.5 mL of Rotieco plus scintillation cocktail (Carl Roth, Karlsruhe, Germany) in a Tri-Carb 2910 TR scintillation counter (Perkin Elmer, Waltham, MA, USA). To determine the transport kinetics for [^3^H]TC in HEK293 cells overexpressing wt and mutant NTCP as well as in Flp-In HEK293 cells (negative control), cells were incubated for 3 min with increasing concentrations (10, 25, 50, 100, 200 and 300 µM) of [^3^H]TC and further transport experiment were conducted on three separate days in triplicates as described above.

### 2.7. Myr-PreS1 Peptide Binding Assay

To study the HBV receptor function of NTCP, HEK293 cells expressing the wt or mutant NTCP constructs were incubated with the N-terminally myristoylated and C-terminally Alexa 568 fluorophore-coupled myr-preS1 viral peptide (further referred to as myr-preS1-AX568), consisting of amino acids 2–48 of the large HBV sub-genotype D3 surface protein (Biosynthesis, Lewisville, TX, USA) [16]. Briefly, cells were washed three times with DMEM and incubated with 50 nM myr-preS1-AX568 peptide in DMEM for 30 min at 37 °C. After extensive washing, cells were fixed with 2% PFA (Carl Roth, Karlsruhe, Germany), washed, and blocked with 5% BSA in PBS for 1 h at room temperature (RT). To study NTCP expression and localization, polyclonal rabbit anti-FLAG antibody (1:500, Sigma-Aldrich, Hamburg, Germany, Cat. #F7425) was incubated at 4 °C overnight, followed by goat anti-rabbit IgG Alexa Fluor 488 (1:1000, Thermo Fisher Scientific, A32731TR) and nuclear staining with Hoechst 33342 (1 mg/mL) for 1 h at RT. Myr-preS1-AX568 peptide binding was analyzed by Leica DMI6000 B inverted fluorescent microscope and LAS X software (Leica, Wetzlar, Germany).

### 2.8. HBV Infection of HepG2-NTCP Cells

HBV was produced in vitro as reported before [16]. HepG2 cells were inoculated with 50,000 genome equivalents of HBV particles per cell for 16 h. For infection experiments, hepatocyte growth medium (HGM) was used, consisting of William’s E Medium (Thermo Fisher Scientific, Waltham, MA, USA) supplemented with 2% bovine serum albumin (Carl Roth, Karlsruhe, Germany), 2 mM L-glutamine (Thermo Fisher Scientific, Waltham, MA, USA), 100 μg/mL gentamicin (Thermo Fisher Scientific, Waltham, MA, USA), 10 nM dexamethasone, 1 mM sodium pyruvate (Thermo Fisher Scientific, Waltham, MA, USA), and 1× Insulin-Transferrin-Selen (Thermo Fisher Scientific, Waltham, MA, USA). During the 16 h infection period, 2% DMSO (Merck, Darmstadt, Germany), 4% polyethylene glycol (PEG) 8000, a mix of antibiotics and antimycotics as well as 100 ng/mL human epidermal growth factor (EGF; Peprotech, Cranbury, NJ, USA) were added as reported [16]. Cells were maintained with HGM lacking PEG and EGF. The medium was changed every three days until fixation at day 12 post infection. HBeAg was measured from 150 µL of cell culture supernatants of HBV infected cells at day 12 post infection were subjected to the qualitative HBeAg Architect assay (Abbott, Wiesbaden, Germany).

### 2.9. Modelling of Human NTCP

The cryo-EM structure of human NTCP (PDB: 7PQQ) [14] was visualized with Protean 3D DNASTAR Software. Visual representation of fluctuation in protein structure and interatomic interactions at amino acid position 146 of wt and mutant NTCP proteins was performed using DynaMut2 server based on the AlphaFold model of human NTCP (AF-Q14973-F1) [26,28].

### 2.10. Statistics

Data are shown as means ± SD. Prism software (GraphPad Prism 6.0, San Diego, CA, USA) was used for data presentation and statistical analysis. Statistical analysis was performed by one-way or two-way analysis of variance (ANOVA) followed by Dunnett’s multiple comparison post-hoc test as indicated in the figure legends. For IC_50_ calculations, statistical analysis was done by two-way ANOVA and Sidak’s multiple comparisons post-hoc test. A *p*-value of <0.001 was considered statistically significant.

## 3. Results

### 3.1. The NTCP 139YIYSRGIY146 Motif Is Crucial for PreS1-Peptide Binding 

**Figure 1:**
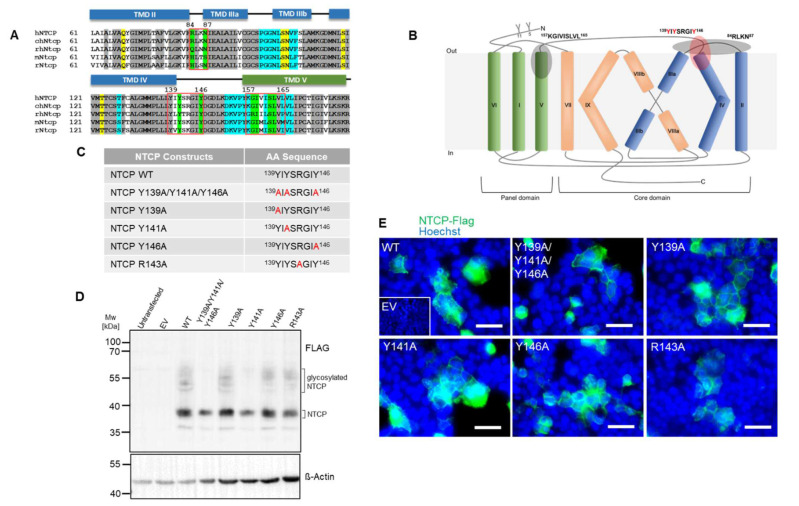
Identification of the tyrosine-enriched motif 139YIYSRGIY146 within human NTCP and characterization of tyrosine-to-alanine NTCP mutants. (**A**) Multiple sequence alignment of human NTCP (hNTCP; Uniprot: Q14973), chimpanzee Ntcp (chNtcp; Uniprot: H2Q8J0), rhesus monkey Ntcp (rhNtcp; Uniprot: F6YRK3), mouse Ntcp (mNtcp; Uniprot: O08705) and rat Ntcp (rNtcp, Uniprot: P26435) using EBI ClustalW algorithm. NTCP sequences that belong to TMD II-IV (blue boxes), TMD V (green box) and loops (black lines) are indicated. Grey shading points out evolutionarily conserved amino acid residues. Red boxes indicate important contact sites at NTCP for the viral preS1 peptide, including the 139YIYSRGIY146 motif analyzed in the present study. Putative residues interacting with the co-substrate Na^+^ are highlighted in yellow. Residues forming the proposed substrate binding pore are highlighted in light blue. Key residues important for preS1 peptide binding (according to [3,11,13,14,15]) are highlighted in green. (**B**) Membrane topology of human NTCP with nine transmembrane domains based on the recent cryo-EM structures of human NTCP (PDB: 7PQG, 7PQQ, 7VAD, 7VAG, 7WSI, and 7FCI). Transmembrane domains are labeled with Roman numbers and NTCP sequences are colored according to (**A**): TMDs I, V, and VI green; II, III, and IV blue; VII, VIII, and IX orange. The positions of the proposed preS1 binding motifs 84RLKN87, 157KGIVISLVL165, and 139YIYSRGIY146 are highlighted. “Y” indicates N-glycosylation at the positions N5 and N11 of NTCP. The proposed panel and core domains are indicated. (**C**) List of generated NTCP mutants with the substituted amino acid residues marked in red. (**D**) Protein expression of the NTCP mutant proteins in HEK293 cells as demonstrated by Western blotting using anti-FLAG antibody. Expression of beta-actin served as loading control. MW, molecular weight; EV, empty vector. (**E**) Microscopy pictures of HEK293 cells transiently transfected with NTCP wt and mutant constructs. NTCP expression was detected using primary anti-FLAG and Alexa Fluor 488-labeled goat anti-rabbit secondary antibodies (green). Nuclei were stained by Hoechst 33342 (blue). Scale bars: 40 µm. All data are representative of three independent experiments.

Inspection of the primary sequence of the human NTCP protein revealed a tyrosine-rich motif YIYSRGIY positioned between amino acids 139–146. Multiple sequence alignment analysis revealed that all three tyrosine residues of this motif, namely Y139, Y141, and Y146, are evolutionarily conserved among human NTCP, as well as chimpanzee, rhesus monkey, rat, and mouse Ntcps (Figure 1A). Based on the recent cryo-EM structures of human NTCP [13,14,15], the 139YIYSRGIY146 motif is localized at the transition of transmembrane domain 4 to extracellular loop 2, and thereby is exposed to the extracellular surface of NTCP that is relevant for preS1-peptide binding (Figure 1B).

In order to address the importance of the 139YIYSRGIY146 motif for regulating the bile acid transport and HBV receptor function of NTCP, single mutants (Y139A, Y141A, R143A and Y146A) and a triple mutant of all three tyrosine residues (Y139A/Y141A/Y146A) were generated by site-directed mutagenesis (Figure 1C). Of note, R143A mutation was included as additional control. As demonstrated by Western blotting (Figure 1D) and microscopic analysis (Figure 1E) of HEK293 cells overexpressing the respective NTCP mutants, all NTCP variants exhibited comparable apparent molecular weights (approximately 38 kDa for the non-glycosylated and ~55 kDa for the glycosylated form) and plasma membrane localization when compared to wt NTCP. However, the NTCP mutants Y139A/Y141A/Y146A and Y141A clearly showed diminished glycosylation. This suggests that tyrosine 141 is important for proper N-glycosylation of NTCP. Noteworthy is the fact that this lack of N-glycosylation did not affect plasma membrane trafficking and localization of the NTCP Y139A/Y141A/Y146A and Y141A mutant proteins (Figure 1E). 

**Figure 2:**
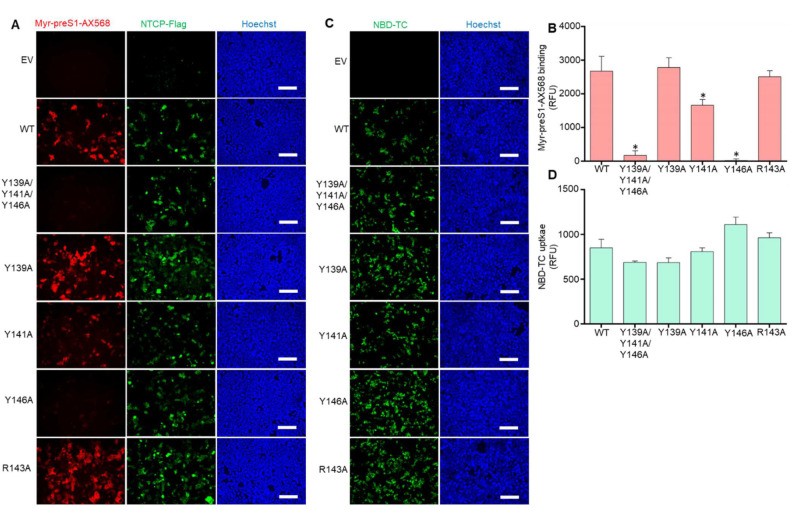
Bile acid transport function and myr-preS1 receptor function of the NTCP mutants. (**A**) Binding of the fluorescently labeled viral myr-preS1-AX568 peptide (red, 50 nM incubated for 20 min at 37 °C) was analyzed using florescence microcopy in HEK293 cells expressing the indicated NTCP mutant constructs. Successful NTCP transfection was confirmed using rabbit anti-FLAG primary and Alexa Fluor 488-labeled goat anti-rabbit secondary antibodies (green). Nuclei were stained by Hoechst 33342 (blue). Scale bars: 130 µm. Representative fluorescence images are shown. (**B**) For quantification of myr-preS1-AX568 peptide binding to NTCP, fluorescence images were captured on Leica DMI6000 B fluorescent microscope and fluorescence signals were quantified with the LAS-X imaging software (Leica) by determining cell-based fluorescence in defined regions of interest (ROI). Data are presented as mean background-subtracted relative fluorescence units (RFU). (**C**) Bile acid transport activity of HEK293 cells overexpressing wt NTCP or the indicated mutant NTCP proteins was analyzed using 5 µM NBD-TC incubated as NTCP substrate for 30 min at 37 °C (green). Nuclei were stained by Hoechst 33342 (blue). Scale bars: 130 µm. (**D**) For quantification of NBD-TC transport into NTCP-expressing HEK293 cells, fluorescence images were quantified with the LAS-X imaging software (Leica) by determining cell-based fluorescence in defined ROI. Data are presented as mean background-subtracted relative fluorescence units (RFU). Data represent means ± SD for three independent experiments. EV, empty vector. * Significantly lower compared to wt NTCP with *p* < 0.001 according to one-way ANOVA with Dunnett’s multiple comparison post-hoc test.

To analyze the effect of NTCP mutation on the preS1-binding ability to NTCP, HEK293 cells overexpressing NTCP wt and mutant proteins were incubated with the fluorescently labeled viral myr-preS1-AX568 peptide. Myr-preS1-AX568 binding to NTCP was visualized using fluorescence microscopy and revealed strong binding to wt NTCP-expressing HEK293 cells, while no binding was observed to empty vector (EV) transfected control cells (Figure 2A). Interestingly, myr-preS1-AX568 binding was completely abolished in the Y139A/Y141A/Y146A triple tyrosine NTCP mutant. More detailed analysis of this effect revealed that most likely Y146A mutation blocked myr-preS1-AX568 binding, while Y139A fully retained, and Y141A at least partly preserved myr-preS1-AX568 binding to the respective NTCP mutant proteins (Figure 2B). In the same way, R143A mutation was fully binding competent for the myr-preS1-AX568 peptide. Next, it was analyzed whether mutations in the 139YIYSRGIY146 motif also affected the bile acid transport function of NTCP. For this, qualitative bile acid transport assays with fluorescently labeled TC were performed (Figure 2C,D). These experiments showed that the bile acid transport function was almost unaffected by any of the NTCP mutations. Noteworthy is the fact that HEK293 cells expressing the R143A NTCP mutation showed identical behavior as wt NTCP, regarding glycosylation (Figure 1D), sorting (Figure 1E), myr-preS1-AX568 peptide binding (Figure 2A,B), and bile acid transport (Figure 2D).

### 3.2. The Role of Y146 and Y141 in the Transporter and Receptor Function of NTCP

**Figure 3:**
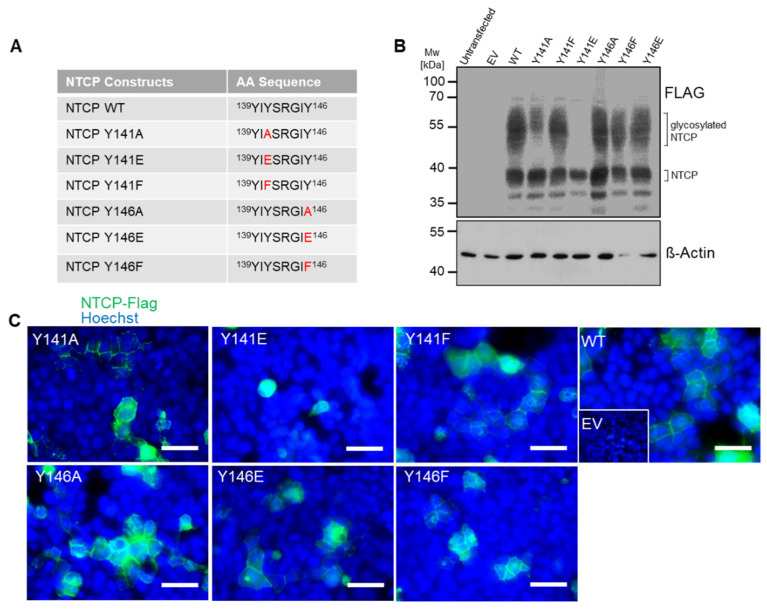
Relevance of tyrosine residues 141 and 146 for the transporter and virus receptor functions of human NTCP. (**A**) List of generated NTCP mutants with the substituted amino acid residues marked in red. (**B**) Protein expression of the NTCP wt and mutants in HEK293 cells was demonstrated by Western blotting using an anti-FLAG antibody. Expression of beta-actin served as loading control. MW, molecular weight; EV, empty vector. (**C**) Microscopy pictures of HEK293 cells overexpressing the NTCP mutant proteins. NTCP expression was detected using primary rabbit anti-FLAG and Alexa Fluor 488-labelled goat anti-rabbit secondary antibodies (green). Nuclei were stained by Hoechst 33342 (blue). Scale bars: 40 µm. All data are representative of three independent experiments.

**Figure 4:**
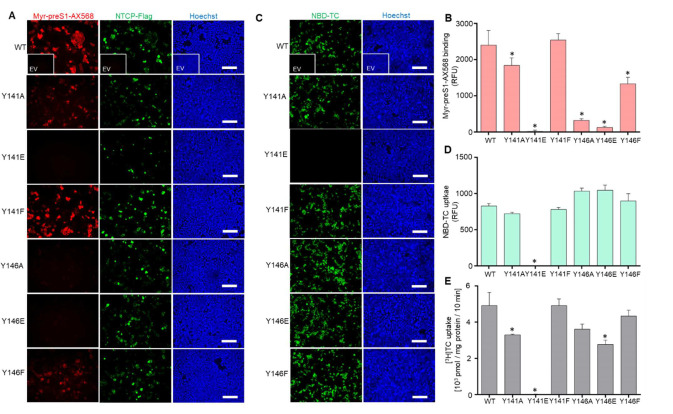
Bile acid transporter function and HBV/HDV myr-preS1 receptor function of the tyrosine 141 and 146 NTCP mutants. (**A**) Binding of the fluorescently labeled viral myr-preS1-AX568 peptide (red, 50 nM incubated for 20 min at 37 °C) was analyzed using florescence microcopy in HEK293 cells transiently transfected with the indicated NTCP mutant constructs. Successful NTCP-expression was confirmed by using rabbit anti-FLAG primary and Alexa Fluor 488-labelled goat anti-rabbit secondary antibodies (green). Nuclei were stained by Hoechst 33342 (blue). Scale bars: 130 µm. Representative fluorescence images are shown. (**B**) For quantification of myr-preS1-AX568 peptide binding to NTCP, fluorescence images were captured on Leica DMI6000 B fluorescent microscope and fluorescence signals were quantified with the LAS-X imaging software (Leica) by determining cell-based fluorescence in defined regions of interest (ROI). Data are presented as mean background-subtracted relative fluorescence units (RFU). (**C**) Bile acid transport activity of HEK293 cells overexpressing wt NTCP and the indicated mutant NTCP proteins was analyzed using 5 µM NBD-TC incubated as NTCP substrate for 30 min at 37 °C (green). Nuclei were stained by Hoechst 33342 (blue). Scale bars: 130 µm. (**D**) For quantification of NBD-TC transport into NTCP-expressing HEK293 cells, fluorescence images were quantified with the LAS-X imaging software (Leica) by determining cell-based fluorescence in defined regions of interest (ROI). Data are presented as mean background-subtracted relative fluorescence units (RFU). (**E**) Quantitative analysis of bile acid uptake into HEK293 cells overexpressing wt and mutant NTCP proteins by using radioactive-labelled [^3^H]TC. Data represent means ± SD for three independent experiments. EV, empty vector. * Significantly lower compared to wt NTCP with *p* < 0.001 according to one-way ANOVA with Dunnett’s multiple comparison post-hoc test.

Tyrosine phosphorylation is a frequently occurring posttranslational protein modification that may regulate protein activity, protein-protein interactions, substrate affinity, and transporter function in SLC transporters [29]. Phosphorylation at tyrosine residues significantly changes the physiochemical properties of these residues by introducing a negative charge at this position of the protein. Another typical feature of tyrosine residues is given by their aromatic ring. Prediction analysis of potential phosphor-motifs within human NTCP using “PhosphoMotif Finder” (http://www.hprd.org; accessed on 20 May 2020) [30,31] revealed that two tyrosine residues at positions 141 and 146 might be potential substrates for ALK, JAK2 or Src kinases. Hence, in order to test whether phosphorylation, and thereby introduction of a negative charge (Y to E mutants), or just the presence of an aromatic ring (Y to F mutants) at positions Y141 and Y146 are relevant for myr-preS1 peptide binding, several additional mutations were introduced into the NTCP protein. To mimic the negative charge at these positions, the tyrosine-to-glutamate mutants Y141E and Y146E were generated and to elucidate the role of the aromatic ring, the tyrosine-to-phenylalanine mutants Y141F and Y146F were made (Figure 3A). All NTCP constructs were expressed in HEK293 cells, and Western blot analysis confirmed expression of the respective NTCP variant proteins. All mutants showed Western blot staining comparable to the wt NTCP, with the exception of the mutant Y141E that demonstrated a lack of N-glycosylated forms around 55 kDa (Figure 3B). In addition, mutant Y141E was the only one that did not present clear expression and plasma membrane staining when transiently expressed in HEK293 cells (Figure 3C). In NTCP-Y141E transfected HEK293 cells, protein expression of this mutant was only scarcely detected with the anti-FLAG antibody (Figure 4A). Consequently, myr-preS1-AX568 peptide binding (Figure 4A,B) as well as NBD-TC (Figure 4C,D) and [^3^H]TC (Figure 4E) transport were completely abrogated by the Y141E mutation of NTCP. In contrast, most of the other NTCP mutants, i.e., Y141A, Y141F, Y146A, Y146E, and Y146F demonstrated the correct plasma membrane trafficking (Figure 4A) and fully active bile acid transport function, as compared to wt NTCP (Figure 4C–E). Interestingly, the Y146A and Y146E mutations of NTCP, although transport positive, were completely binding negative for the myr-preS1-AX568 peptide (Figure 4A,B). However, substitution of Y146 with phenylalanine slightly recovered the myr-preS1-AX568 peptide binding competence of NTCP to an intermediate level when compared with wt NTCP and the Y146E/Y146A mutants (Figure 4A,B). Another interesting finding was that, although Y141E mutation negatively affected NTCP expression and function, Y141F modification revealed effects similar to wt NTCP. This replacement fully retained bile acid transport and myr-preS1-AX568 peptide binding, indicating that the aromatic ring of the phenylalanine residue at this position is sufficient for both functions of NTCP.

### 3.3. Y146A, Y146E, and Y146F Mutants Show Similar Transport Kinetics with WT NTCP

**Figure 5:**
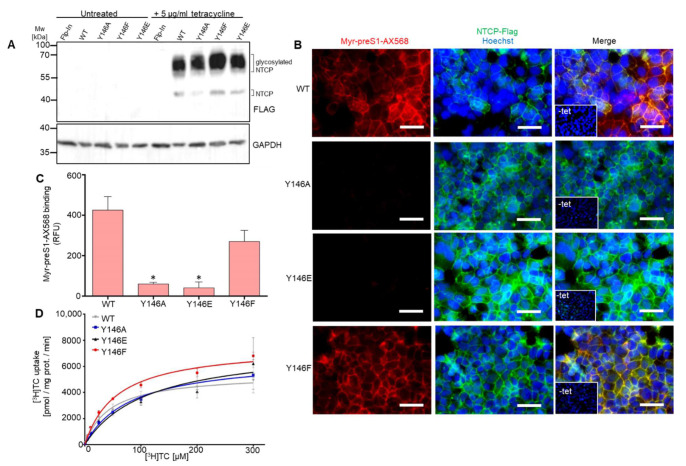
TC transport kinetics in stably NTCP-transfected HEK293 cell lines. In addition to wt NTCP, the cDNA constructs of the Y146A, Y146E, and Y146F mutants were stably transfected into the tetracycline-inducible T-Rex Flp-In HEK293 cells and single cell clones were selected. (**A**) NTCP protein expression in the stably transfected cell lines with or without addition of tetracycline (5 µg/mL) for 48 h. Cell lysates were processed for Western blot analysis using an anti-FLAG antibody. Expression of GAPDH served as loading control. MW, molecular weight marker. (**B**) Binding of the myr-preS1-AX568 peptide (red) to HEK293 cells stably expressing the wt NTCP, as well as the NTCP Y146A, Y146E, and Y146F mutants. The NTCP protein was counterstained with the rabbit anti-FLAG primary and the Alexa Fluor 488-labelled goat anti-rabbit secondary antibodies (green). Nuclei were visualized with Hoechst dye (blue). Scale bars: 40 µm. (**C**) For quantification of myr-preS1-AX568 peptide binding to NTCP, fluorescence images were captured on Leica DMI6000 B fluorescent microscope and fluorescence signals were quantified with the LAS-X imaging software (Leica) by determining cell-based fluorescence in defined regions of interest (ROI). Data are presented as mean background-subtracted relative fluorescence units ± SD of three independent experiments. EV, empty vector. * Significantly lower compared to wt NTCP with *p* < 0.001 according to one-way ANOVA with Dunnett’s multiple comparison post-hoc test. (**D**) Concentration-dependent uptake of [^3^H]TC was analyzed in HEK293 cells stably expressing the wt NTCP, as well as the NTCP Y146A, Y146E, and Y146F mutants. Cells were treated with 5 μg/mL tetracycline for 48 h to induce carrier expression. Non-transfected T-Rex Flp-In HEK293 cells served as control. Specific uptake of [^3^H]TC was calculated by subtracting non-specific uptake of the T-Rex Flp-In control cells from uptake into carrier-overexpressing HEK293 cells. The values represent means ± SD of three independent experiments.

Although the NTCP mutants Y146A and Y146E were binding negative for the myr-preS1-AX568 peptide, the bile acid transport function was completely preserved when TC uptake experiments were performed with NBD-TC and [^3^H]TC in transiently transfected HEK293 cells (Figure 4C,D). However, as the total amount of expressed protein can vary to some degree after transient transfection of different cDNA clones and, in addition, as these transport experiments were only performed at one single substrate concentration, small differences in the transport behavior of a mutant carrier can easily be overseen. To get around this disadvantage, we additionally established cell lines stably transfected with the NTCP variants Y146A, Y146E, and Y146F. Expression of the mutant NTCP proteins was analyzed by Western blotting and revealed similar signal intensities as compared to wt NTCP. Of note, in the stably transfected cell lines, the glycosylated form dominated in the Western blot analysis at an apparent molecular weight of ~55 kDa, whereas only faint bands were detected for the suspected non-glycosylated form at ~38 kDa (Figure 5A). In addition, protein expression and sorting were analyzed in the stably transfected cell lines by immunofluorescence microscopy after staining of the FLAG-tagged NTCP proteins with a respective antibody. As shown in Figure 5B, wt NTCP as well as the NTCP mutants Y146A, Y146E, and Y146F were clearly detectable at the plasma membrane. Binding experiments with the myr-preS1-AX568 peptide confirmed the strong association to the wt NTCP. In contrast, the myr-preS1-AX568 peptide did not bind to the cell surface of the HEK293 cells stably expressing the NTCP Y146A and Y146E mutants. In the case of the Y146F NTCP mutant, binding of the myr-preS1-AX568 peptide could be detected, however, at lower level compared to wt NTCP (Figure 5C). The stable cell lines were also used to measure the full TC transport kinetics by using increasing substrate concentrations. As shown in Figure 5D, wt NTCP, and the mutants Y146A, Y146E, and Y146F showed comparable transport kinetics with *K*_m_ values of 44.8 ± 8.9, 82.4 ± 5.9, 112.4 ± 37.4, and 57.3 ± 5.6 µM and *V*_max_ values of 5444 ± 336, 6683 ± 198, 7618 ± 1067, and 7579 ± 248 pmol/mg protein/min, respectively.

### 3.4. NTCP Y141E, Y146A and Y146E Mutations Protect against In Vitro HBV Infection

**Figure 6:**
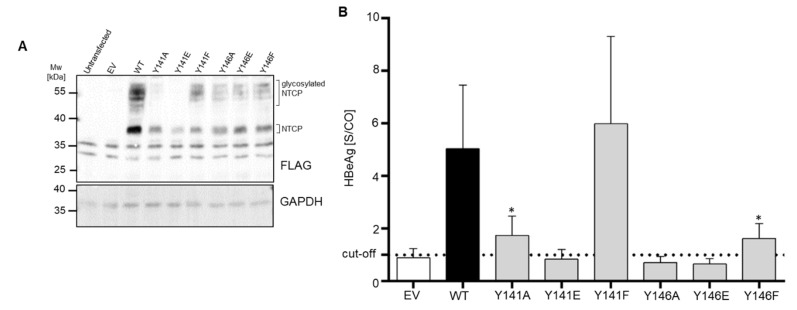
In vitro HBV infection of HepG2 cells expressing wt NTCP or the NTCP mutants Y141A, Y141E, Y141F, Y146A, Y146E, and Y146F. HepG2 cells were transiently transfected with the indicated constructs. (**A**) Protein expression of the NTCP wt and mutants in HepG2 cells as demonstrated by Western blotting using anti-FLAG antibody. Expression of GAPDH served as loading control. MW, molecular weight; EV, empty vector. (**B**) Forty-eight h after transfection, cells were infected with HBV for 16 h. At 12 days post infection, cell culture supernatants of HBV infected cells were collected and de novo production of HBeAg was qualitatively evaluated in a commercial HBeAg assay. Data represent means ± SD of three independent experiments. S/CO, signal-to-cut-off ratio. EV, empty vector. * Significantly lower compared to wt NTCP with *p* < 0.001 according to one-way ANOVA with Dunnett’s multiple comparison post-hoc test.

In order to test whether mutations of the 139YIYSRGIY146 region would have any effect on HBV susceptibility, wt and mutant NTCP constructs were transiently transfected into HepG2 cells and further utilized for in vitro HBV infection studies. Western blot analysis confirmed successful expression of all NTCP mutant proteins in HepG2 cells (Figure 6A). The rate of infection of HepG2 cells was analyzed by HBeAg secretion. For this assay, a signal-to-cut-off ratio (S/CO) of more than one indicated successful HBV infection (Figure 6B). HepG2 cells overexpressing wt NTCP, as well as the NTCP Y141F mutant, showed equal susceptibility for in vitro HBV infection. In contrast, HepG2 cells expressing the Y141E, Y146A, and Y146E mutants did not support HBV infection at all. In the case of the NTCP mutants Y141A and Y146F, HBeAg secretion could be detected, however, at a significantly lower level compared to wt NTCP. 

## 4. Discussion

**Figure 7:**
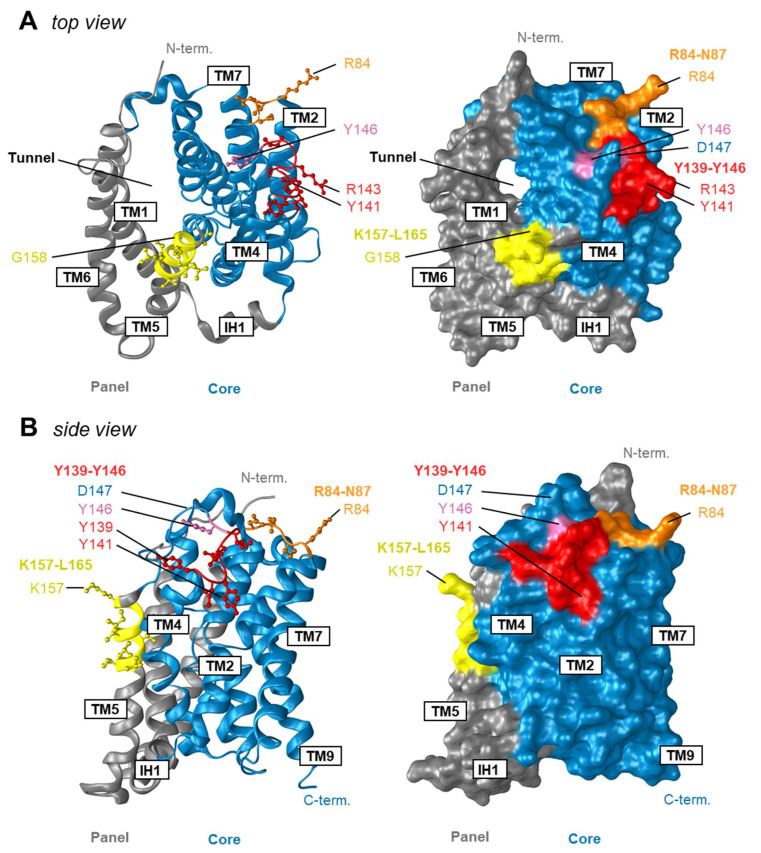
Regions at the human NTCP that are critical for viral myr-preS1 peptide binding. The structure is based on thermostabilized human NTCP in complex with Megabody 91 (PDB entry: 7PQQ; [14]) and demonstrates the localization of the Y141- and Y146-containing 139YIYSRGIY146 NTCP motif (red) in top view (**A**) and side view (**B**). Amino acid residue Y146 is labeled in pink. The model was visualized with the Protean 3D DNASTAR Software. Positions of amino acids of human NTCP involved in viral myr-preS1 peptide binding are labeled with colors: 157KGIVISLVL165 (yellow) and 84RLKN87 (orange).

To date, little is known about the mechanistic insights into the process of initial NTCP-HBV complex assembly and NTCP-mediated virus entry into hepatocytes [9,10]. Although, during revision of the manuscript, three independent cryo-EM structures of human NTCP were published [13,14,15], partly with bound viral myr-preS1 peptide, the precise molecular interactions between the host-derived NTCP receptor and the HBV surface proteins are still not fully understood. Experimental approaches, such as protein engineering and computational biology methods, are powerful tools in exploring motifs within the NTCP protein that are essential for regulation of its HBV affinity during infection [3,9,10,12,23,24,32,33]. In this study, we identified a novel NTCP tyrosine-enriched motif 139YIYSRGIY146 that is significantly involved in the regulation of NTCP-HBV preS1 interactions and, thus, in the control of NTCP receptor function (Figure 7A,B). The most striking findings of the presented data are: (1) the novel preS1-binding-motif 139YIYSRGIY146 contains evolutionarily conserved Y139, Y141 and Y146 aa residues; (2) tyrosine residue Y146, and to some extent Y141, were critical for the preS1-binding to NTCP and viral infection; (3) the aromatic ring of Y146 may play a critical role in intramolecular hydrophobic interactions between K153, Y146 and N87 and is critical for virus entry; (4) the hydroxyl group of Y146, but not that of Y141, seems to directly interact with the viral myr-preS1 peptide; and, finally, (5) specific mutations in the 139YIYSRGIY146 motif preserved the transporter function of NTCP, pointing to the tyrosine-enriched motif as a promising target for the development of potent HBV entry inhibitors.

**Figure 8:**
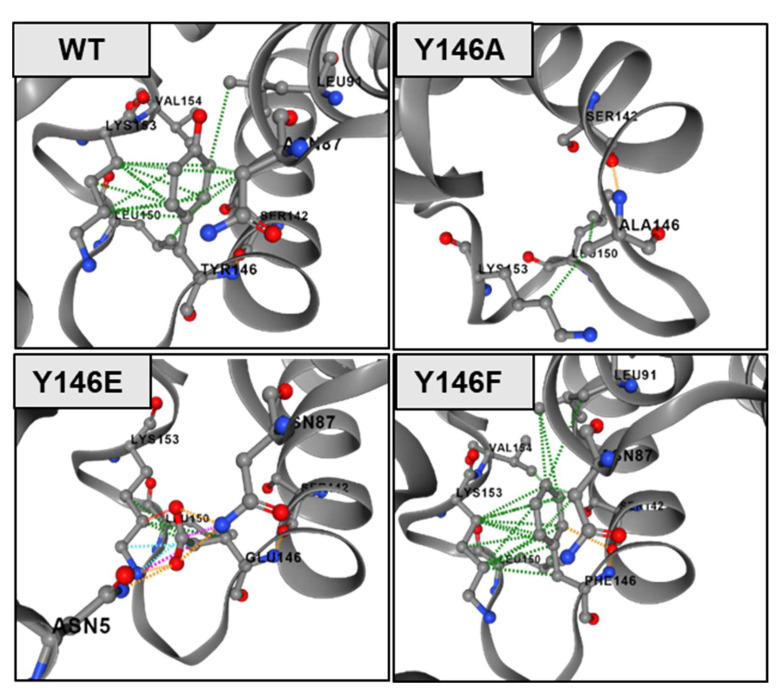
Visual representation of interatomic interactions at amino acid position 146 of wt and mutant NTCP proteins. Hydrophobic interactions are depicted as dotted green lines, ionic interaction in orange and steric clashes (unnatural overlap of two non-bonding atoms) in pink based on the AlphaFold structure of human NTCP (AF-Q14973-F1).

In mutation studies, effects of loss-of-function mutants are often difficult to interpret, as even single amino acid mutations can destabilize a protein in a way that ligand or substrate binding is no longer possible. However, there is, of course, also the possibility for a direct interaction of a particular amino acid residue with the above-mentioned molecules so that a loss of function phenotype can be explained by lack of a specific interaction site. In the case of the Y141E NTCP mutant, in the present study, this mutation seems to destabilize the NTCP protein in a way that means sufficient protein expression and sorting to the plasma membrane is not possible. Consequently, this mutant is no longer active in bile acid transport and myr-preS1-AX568 peptide binding (Figure 3B,C, and Figure 4A,C). In contrast, all other mutants showed fully active bile acid transport functions, clearly indicating that the respective mutations did not affect protein expression and stability, as well as protein flexibility, that are important to execute conformational changes during the transport cycle. Nevertheless, it cannot be excluded that, even if bile acids and myr-preS1 peptide binding to NTCP significantly interfere with each other [16,34], a mutation might destabilize the NTCP protein in a way that only myr-preS1 peptide binding is affected, but not bile acid transport. Considering this, it is interesting to note that the NTCP Y141F mutant retained the same full transport and virus receptor function as the wt NTCP, indicating that the hydroxyl group of the tyrosine at amino acid position 141 is neither involved in the binding of the bile acid nor of the myr-preS1 peptide. This fits quite well with the localization of Y141 within the recently resolved NTCP structure, where this tyrosine residue is clearly oriented away from the domains relevant for substrate and preS1 binding (Figure 7A,B). Nevertheless, the aromatic ring at this amino acid position seems to be important for the conformation and stability of the NTCP protein. Accordingly, substitution of tyrosine with alanine at position 141 seems to change the structure of the whole NTCP protein in a way that bile acid transport (Figure 4E), myr-preS1-AX568 peptide binding (Figure 4B), and in vitro HBV infection (Figure 6B) are reduced. The situation was somewhat different for mutations at amino acid position Y146. The tyrosine residue Y146 seems to form significant hydrophobic interactions with the adjacent lysine 153 (K153) and asparagine 87 (N87) residues, at least based on the AlphaFold-derived NTCP model (Figure 8). Here, Y146 is located exactly at a bridge region connecting the K157-L165 and R84-N87 motifs that were previously identified as relevant regions for HBV/HDV virus binding to NTCP (Figure 7A,B). This might explain why mutation of Y146 to alanine, possibly abolishing the K153-Y146-N87 hydrophobic interaction axis, comes with a loss of myr-preS1 peptide binding phenotype (Figure 8, panel WT vs. Y146A). Interestingly, the hydroxyl group of tyrosine 146 seems to make no interaction with any of the surrounding amino acid residues, suggesting that the Y146F mutation does not affect stability and folding of the NTCP protein. This is supported by the fact that the transport kinetics for TC are nearly identical to those for the wt NTCP and the Y146F mutant (Figure 5C). However, we demonstrated markedly lower binding of the myr-preS1-AX568 peptide to the Y146F mutant (Figure 4B) compared to wt NTCP and, as a consequence, significantly reduced rates for in vitro HBV infection (Figure 6B). Overall, this might indicate that the hydroxyl group of the tyrosine 146 residue is directly involved in the interaction with the myr-preS1 peptide, probably via hydrogen bonding. However, additional studies are needed to analyze the contribution of these non-covalent interactions of Y146 to the preS1 binding activity of NTCP upon HBV infection.

Protein surface-exposed regions of NTCP play an essential role during initial docking of HBV to hepatocytes [35]. Until now, only few such motifs have been identified and characterized giving prerequisites for the prediction of preS1-NTCP interactions taking place upon HBV infection [6,9,10]. It seems that different NTCP motifs are required for high-affinity virus binding and entry into hepatocytes, such as 84RLKN87, 157KGIVISLVL165, S267, and N5/N11 [6,9,10]. According to recent NTCP models, the protein possesses nine transmembrane helices, which are arranged into a “core” domain composed of TMDs II, III, IV, VII, VIII, and IX, and a “panel” domain comprised of TMDs I, V and VI (Figure 1B and Figure 7A,B). The newly identified 139YIYSRGIY146 is part of the second extracellular loop linking TMD IV and TMD V [10,13,14,15,36,37,38]. Therefore, the Y146 residue lies near previously identified sequences required for HBV tropism, namely 157KGIVISLVL165 and 84RLKN87, which are located in the TMD V, and in the first extracellular loop between TMD II and TMD III, respectively [10,13,14,15]. Their close proximity and protein surface localization suggest a joint contribution to virus binding. Since the Y146-containing extracellular loop connects TMD IV and TMD V, thereby bridging the “core” and “panel” domains of NTCP [10,13,14,15], it was proposed that a movement of the more flexible “panel” domain (TMD I, V, VI) against the more rigid “core” domain (TMD II–IV and VII–IX) facilitates bile acid transport of NTCP [10,13,14,15,37,39]. Consequently, any mutation in the 139YIYSRGIY146-connecting loop of these two domains should significantly affect protein structure and conformation. Subsequently, this could result in abnormal transporter function. Surprisingly, except for the “loss-of function” Y141E variant, all other tested NTCP mutants, including 143K (data not shown), were capable of transporting TC into the HEK293 cells, suggesting that the 139YIYSRGIY146 motif is irrelevant for the transporter function of NTCP. Therefore, the second extracellular loop of NTCP, especially the fragment comprised of Y141 and Y146, could be a potential target for the development of NTCP-based entry inhibitors, which would not disrupt the enterohepatic circulation of bile acid in humans.

## 5. Conclusions

In conclusion, we have provided evidence that the newly identified 139YIYSRGIY146 motif and its Y141 and Y146 aromatic amino acids are important virulence factors that play a fundamental role in the process of NTCP-mediated HBV tropism. Our comprehensive analyses of NTCP Y141 and Y146 mutants confirmed the importance of intramolecular interactions of these aromatic amino acids with previously proposed protein surface-localized NTCP regions involved in HBV tropism, such as 84RLKN87. The current study presents new insights into the HBV-NTCP interaction and further prerequisites for the development of novel HBV entry inhibitors aimed at blocking the initial virus-receptor complex assembly.

## Figures and Tables

**Table 1:** Oligonucleotide primers used for site-directed mutagenesis of NTCP.

Primer	Sequence (5′→3′)
Y139A/Y141A/Y146A *	For: GAT GCC TCT CCT CCT GGC CAT CGC CTC CAG GGG GAT CGC TGA TGG GGA CCT GAA GG Rev: CCT TCA GGT CCC CAT CAG CGA TCC CCC TGG AGG CGA TGG CCA GGA GGA GAG GCA TC
Y139A	For: GAT GCC TCT CCT CCT GGC CAT CTA CTC CAG GGG G Rev: CCC CCT GGA GTA GAT GGC CAG GAG GAG AGA GGC ATC
Y141A	For: CCT CTC CTC CTG TAC ATC GCC TCC AGG GGG ATC TAT GATGGG Rev: CCC ATC ATA GAT CCC CCT GGA GGC GAT GTA CAG GAG GAG AGG
Y141E	For: CCT CTC CTC CTG TAC ATC GAG TCC AGG GGG ATC TAT GAT GGG Rev: CCC ATC ATA GAT CCC CCT GGA CTC GAT GTA CAG GAG GAG AGG
Y141F	For: CCT CTC CTC CTG TAC ATC TTC TCC AGG GGG ATC TAT GAT GGG Rev: CCC ATC ATA GAT CCC CCT GGA GAA GAT GTA CAG GAG GAG AGG
Y146A	For: CAT CTA CTC CAG GGG GAT CGC TGA TGG GGA CCT GAA GGA C Rev: GTC CTT CAG GTC CCC ATC AGC GAT CCC CCT GGA GTA GAT G
Y146E	For: CAT CTA CTC CAG GGG GAT CGA AGA TGG GGA CCT GAA GGA Rev: GTC CTT CAG GTC CCC ATC TTC GAT CCC CCT GGA GTA GAT G
Y146F	For: CAT CTA CTC CAG GGG GAT CTT TGA TGG GGA CCT GAA GGA C Rev: GTC CTT CAG GTC CCC ATC AAA GAT CCC CCT GGA GTA GAT G
R143A	For: CTC CTG TAC ATC TAC TCC GCG GGG ATC TAT GAT GGG GAC Rev: GTC CCC ATC ATA GAT CCC CGC GGA GTA GAT GTA CAG GAG

* The Y139A NTCP mutant construct served as a template for side-directed mutagenesis to obtain the triple mutant Y139A/Y141A/Y146A; For, forward primer; Rev, reverse primer.

## Data Availability

The data that support the findings of this study are available on request from the corresponding author.
